# Anthelmintic efficacy on *Parascaris equorum *in foals on Swedish studs

**DOI:** 10.1186/1751-0147-51-45

**Published:** 2009-11-22

**Authors:** Eva Osterman Lind, Dan Christensson

**Affiliations:** 1 National Veterinary Institute, Department of Virology, Immunobiology and Parasitology, Section for Parasitological Diagnostics, SE-751 89 Uppsala, Sweden

## Abstract

**Background:**

In the last few years stud farms have experienced increasing problems with *Parascaris equorum *infections in foals despite intensive deworming programs. This has led to the question as to whether the anthelmintic drugs used against this parasite are failing. This study aimed to investigate the efficacy of ivermectin, fenbendazole and pyrantel on the faecal output of ascarid eggs of foals.

**Methods:**

A Faecal Egg Count Reduction Test (FECRT) was performed on nine large studs in Sweden. Anthelmintic drugs were given orally and faecal samples were examined for ascarid eggs on the day of deworming and 14 days later. Faecal Egg Count Reductions (FECRs) were calculated on arithmetic means of transformed individual FECRs and on arithmetic means of individual FECRs.

**Results:**

Seventy-nine (48%) out of a total of 165 foals sampled were positive for *P. equorum *eggs before deworming and 66 of these met the criteria for being used in the efficacy assessment. It was shown that there was no, or very low activity of ivermectin on the output of ascarid eggs in the majority of the foals, whereas for fenbendazole and pyrantel it was >90%.

**Conclusion:**

Ivermectin resistance was shown in 5 out of 6 farms. Therefore, ivermectin should not be the drug of choice in the control of *P. equorum *infections in foals. According to the results of this study, fenbendazole or pyrantel are still effective and should be used against this parasite.

## Introduction

*Parascaris equorum *(Nematoda: Ascaridoidea) infections occur commonly in foals and yearlings. The horses ingest infective eggs that are dispersed in the surrounding environment by previous years' foals. Infections may cause nasal discharge, coughing, ceased growth, inappetence, rough hair coat and lethargy. At large worm burdens intestinal obstruction may occur [1]. Immunity against *P. equorum *starts to develop by the age of 6 months [2] and horses older than 4 years seldom harbour these nematodes.

Generally, foals are dewormed against *P. equorum *several times during their first 12 months of life. Anthelmintic drugs registered in Sweden for this purpose include three drug classes: benzimidazoles (fenbendazole and febantel), tetrahydropyrimidines (pyrantel) and macrocyclic lactones (ivermectin and moxidectin). During the last few years it has been noticed on several Swedish stud farms that foals pass mature ascarid worms with faeces shortly after deworming. Also, obstruction and rupture of the intestine have occurred in foals that have been rigorously dewormed according to veterinarians' recommendations. Recently, it was demonstrated that foals on a stud farm excreted high numbers of ascarid eggs despite ivermectin treatment every 8^th ^week [3]. These observations have raised the question as to whether the registered drugs are ineffective against *P. equorum*. In 2000, resistance to macrocyclic lactones in *P. equorum *was suspected on a trotter stud in the Netherlands [4]. Later, the possibility of ivermectin resistance was also reported from North America [5-7], Germany [8] and Denmark [9].

The aim of this field study was to: 1) determine the efficacy of ivermectin, pyrantel and fenbendazole on the faecal output of *P. equorum *eggs passed by foals on 9 big stud farms; and 2) investigate the occurrence of *P. equorum *infections on these studs.

## Materials and methods

A total of 165 foals bred and housed on nine big studs in Sweden were included in a Faecal Egg Count Reduction test (FECRT). The study took place in the autumn of 2005, after weaning (table 1). In eight of the studs the horses were Swedish standardbred racing trotters and in one they were half bred riding horses. All of the studs routinely accommodate external mares during the breeding season. The median age of the foals was 6.5 months and they had been dewormed 2 or 3 times during the summer prior to the study.

**Table 1:** Data on foals/studs sampled in the study.

*Farm* *id*	*Age (wks) at 1*^st^*AH treat*	*No AH treat 1*^st^*year*	*AH used prior to study*	*Number of foals sampled*	*% inf* *P. equorum d 0*
1	8	6	pyr	15	60
2	8	3	iv	19	37
3	6	6	iv	17	47
4	6	7	iv	23	52
5	8	6	fbz	14	43
6	4	5	iv	18	56
7	8	6	iv	19	84
8	1	5	iv	22	41
9	8	6	iv + pyr	18	11
*Overall*				*165*	*48*

Wks - weeks; AH - anthelmintic; treat - treatment; inf - infected; d 0 - the day of AH treatment.

One group of foals on each of 7 studs, and 2 groups on 2 studs, were treated with anthelmintic drugs in the following way: ivermectin (6 groups, Bimectin^®^^1^/Ivomec^®^^2^/Noromectin^®^^3^), fenbendazole (3 groups, Axilur^®^^4^), pyrantel embonate (1 group, Banminth^®^^5^) or ivermectin + pyrantel embonate (1 group, Noromectin^® ^+ Banminth^®^). The weights of the foals were estimated visually by an experienced local veterinarian/horse manager and rounded up to the nearest 50 kg. The veterinarian/manager administered the drugs orally according to the manufacturers' recommendations. A faecal sample was taken from each foal on the day of treatment (day 0) and 14 days later (day 14). These samples were placed in plastic bags, which were sealed and sent to the laboratory of the National Veterinary Institute. The numbers of ascarid and strongyle eggs per gram faeces (EPG) were determined by a modified McMaster technique with a lowest detection level of 50 EPG [10]. Calculations on the efficacy of the anthelmintic drugs were performed on samples that contained 100 EPG or more of *Parascaris equorum *on day 0. On stud No. 9, there were only 2 foals (out of 18) excreting sufficient numbers of eggs.

The foals were distributed into age groups depending on age (months) at the time for sampling. Age differences in the faecal output of eggs were analysed with 1-factor ANOVA (using the SYSTAT v. 5.1 program) on log-transformed values as the data better fitted a lognormal distribution. The Faecal Egg Count Reductions (FECRs) were calculated in two ways: arithmetic means of transformed individual FECRs according to Pook *et. al*. [[11], Method 2], and arithmetic means of individual FECRs. Individual faecal egg count reduction (FECR) was calculated in the following way: FECR = (1-(EPG_d14_/EPG_d0_)) × 100%

## Results

Approximately half (48%) of the 165 foals excreted eggs of *Parascaris equorum *at the first sampling occasion (table 1). Sixty-six of these met the criteria for being included in the FECR calculations. The output of eggs did not differ significantly between foals of different ages (1-factor ANOVA: p = 0.231; 1-factor ANOVA on log-transformed data: p = 0.590).

The FECR following ivermectin treatment varied from 0-100%, with a mean reduction of 50% (transformed values) and 36% (individual FECRs) (table 2). Depending on the calculation method, the lower 95% confidence intervals varied from 0%-20% and 0%-60%, respectively. In all but one stud where ivermectin was used, the FECR was lower than 90%. On studs No. 3, 4 and 6 all the foals included in the study were shedding *P. equorum *eggs 14 days following ivermectin treatment.

**Table 2:** Faecal Egg Count Reduction Test performed on 9 studs.

*Farm*	*AH*	Group size*	*Group mean EPG d 0*	*Group mean EPG d 14*	*Max EPG d 0*	*Max* *EPG d 14*	***FECR***^1^ *d 14*	***FECR***^2^ *d 14*
1	iv	6	525	58	1100	200	78%	85%
2	iv	7	336	0	1350	0	100%	100%
3	iv	7	1157	721	4000	2300	7%	11%
4	iv	7	900	907	2600	1500	2%	0%
5	iv	6	283	125	500	250	24%	56%
6	iv	9	1194	961	3500	2200	1%	0%
								
4	fbz	3	1417	0	3550	0	100%	100%
7	fbz	5	700	0	1000	0	100%	100%
8	fbz	5	1750	0	2900	0	100%	100%
								
7	pyr	9	1133	34	2400	200	94%	93%
9	iv+pyr	2	125	5	150	10	91%	95%

AH - anthelmintic; iv - ivermectin; fbz - fenbendazole; pyr - pyrantel; EPG - number of eggs per gram faeces; mean EPG - arithmetic mean; d 0 - day of anthelmintic treatment; d 14 - 14 days following anthelmintic treatment; FECR - Faecal Egg Count Reduction.

* Only foals with at least 100 EPG included

^1 ^Arithmetic means of transformed individual FECRs (Pook *et al*. 2002)

^2 ^Arithmetic means of individual FECRs

In the fenbendazole treated groups the FECR was 100% and no foals were excreting ascarid eggs 14 days after deworming (table 2). Also, in the 9 horses that had been treated with pyrantel the reduction of eggs was more than 90% (table 2).

## Discussion

The prevalence of *Parascaris equorum *in foals is known to be high, commonly in the range 31-61% [12]. The finding of *P. equorum *eggs in 48% of the samples before anthelmintic treatment was within the range for what could be expected in the autumn.

The most striking result from this study was that in 5 studs out of 6, ivermectin treatment failed to suppress the faecal output of *P. equorum *eggs. Several foals were even excreting higher numbers of ascarid eggs following ivermectin deworming, some of them more than 2000 EPG. Since the same pattern was seen in several farms it is unlikely that under-dosing could explain the low reduction of *P. equorum *eggs. Moreover, no strongyle eggs were found in the samples of the ivermectin-treated individuals, which indicates that the horses had been dosed correctly.

Since the mid 1980s, the ivermectin drugs have been marketed and used successfully to control nematodes in horses. There are several studies published where ivermectin has been shown to have 100% effect against adult stages of *P. equorum *[13-16]. Moreover, anthelmintic resistance in *P. equorum *has been considered to be improbable because a great proportion of the parasite population, the long-living eggs in the environment, are in so called refugia, that is they are not exposed to the drug and thus are not selected for resistance. Still, ivermectin resistance in *P. equorum *has recently been reported from both Europe and North America. The present study clearly shows that ivermectin resistance is now a widespread problem in Swedish stud farms.

Reduced efficacy of ivermectin against *P. equorum *was noticed for the first time in a Swedish stud in 1997. Since then, the development of resistance appears to have accelerated in response to excessive, sometimes exclusive use of ivermectin in foals. For two decades, foals on studs have commonly been treated every 8^th ^week with ivermectin, and over the years this might have resulted in a decreased proportion of susceptible genotypes in refugia. Significant traffic of mares and foals between horse premises has probably contributed to an effective spread of resistant genotypes, as discussed by Reinemeyer [17].

In the 3 groups where fenbendazole was tested the egg reduction was 100%. This was in accordance with a Canadian study [7]. A reduction of 100% following fenbendazole treatment was also obtained in a recent FECRT of 24 Swedish foals (Osterman Lind, unpublished data).

The efficacy of pyrantel was >90%, but since there were only 9 individuals on one farm that received pyrantel, the existence of pyrantel resistance in *P. equorum *cannot be ruled out. However, the Canadian study [7], as well as unpublished Swedish data on 25 foals (Osterman Lind), also showed a high efficacy of pyrantel.

## Conclusion

Ivermectin resistance in *P. equorum *was detected in 5 out of 6 equine farms. Fenbendazole and pyrantel, on the other hand, were both effective in reducing the faecal output of ascarid eggs. On most of the premises included in the study, the deworming practices of foals have now been changed. Instead of ivermectin, fenbendazole or pyrantel are now the drugs of choice for use against *P. equorum*. It is important, however, that the anthelmintic efficacy is monitored routinely by FECRT on these breeding premises. In the long-term it is also necessary to incorporate non-chemotherapeutic methods to a greater extent to control parasite infections on stud farms.

## Competing interests

The authors declare that they have no competing interests.

## Authors' contributions

Both authors designed the study and interpreted the results. EOL drafted the manuscript, which was read and approved by DC.
